# Visceroptosis and the Ehlers-Danlos Syndrome

**DOI:** 10.7759/cureus.1828

**Published:** 2017-11-08

**Authors:** Stephen Kucera, Stephen N Sullivan

**Affiliations:** 1 Patient; 2 Medicine/island Medical Program, University of Victoria, British Columbia, Canada

**Keywords:** ehlers-danlos syndrome, visceroptosis, functional gastrointestinal disorders

## Abstract

The case of a patient with visceroptosis and Ehlers-Danlos syndrome hypermobility type (RDS-HT) is reported here. The literature on this unusual but probably under-recognized complication is reviewed.

## Introduction

The Ehlers-Danlos syndromes (EDS) are a heterogenous group of inherited collagen disorders. They manifest as joint hypermobility, skin hyperextensibility, tissue fragility, and a host of potential symptoms and sometimes very serious complications, many of which are gastrointestinal. We tell the story of a patient with the hypermobility type of EDS (EDS-HT), complicated by severe constipation, gastroparesis, nausea, intractable abdominal pains and visceroptosis - defined as the displacement of various abdominal organs from their normal positions. The scant literature of this unusual but probably under-recognized complication of EDS-HT is reviewed.

## Case presentation

The patient's story

**Figure 1:**
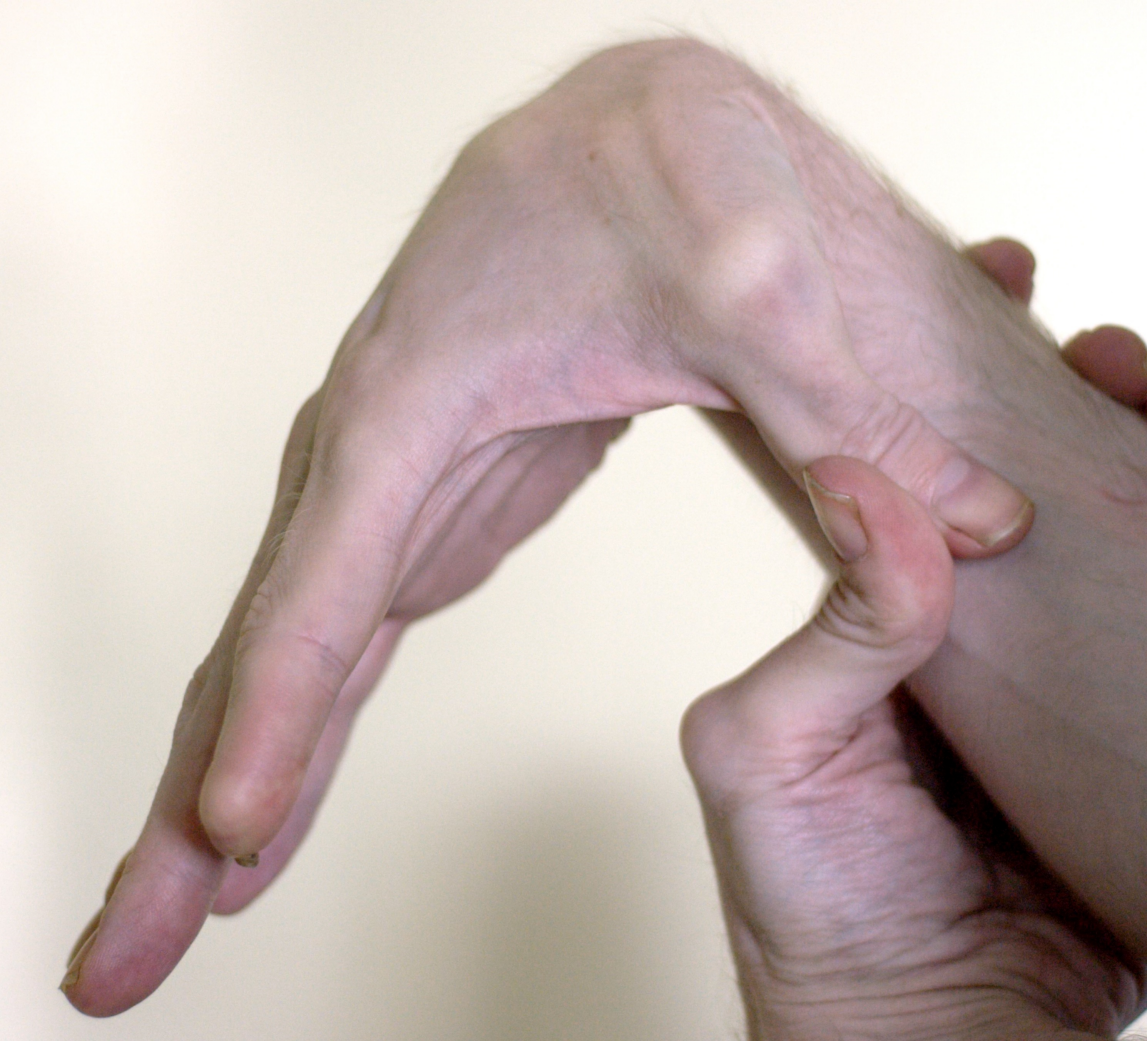
Flexible thumb

**Figure 2:**
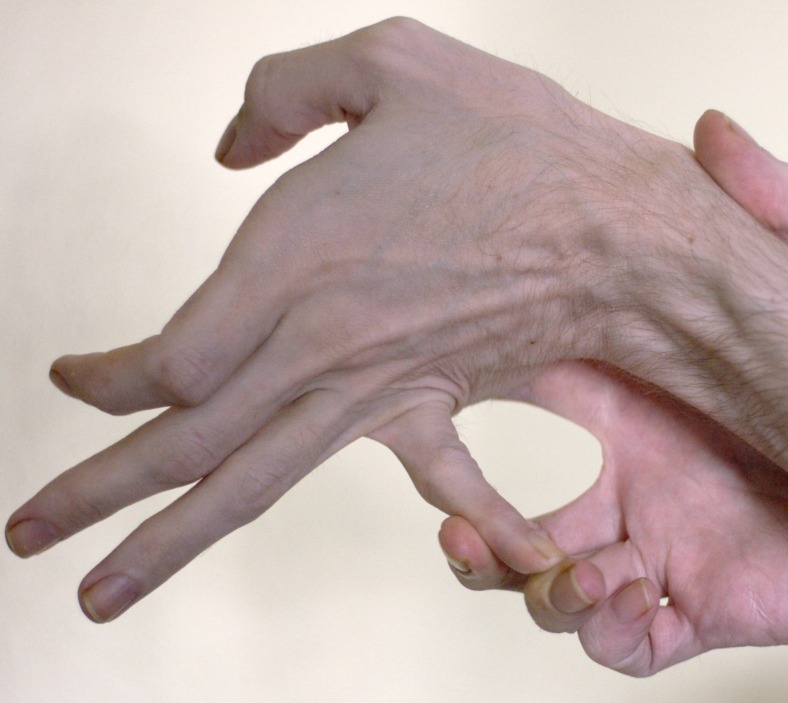
Flexible fifth digit

**Figure 3:**
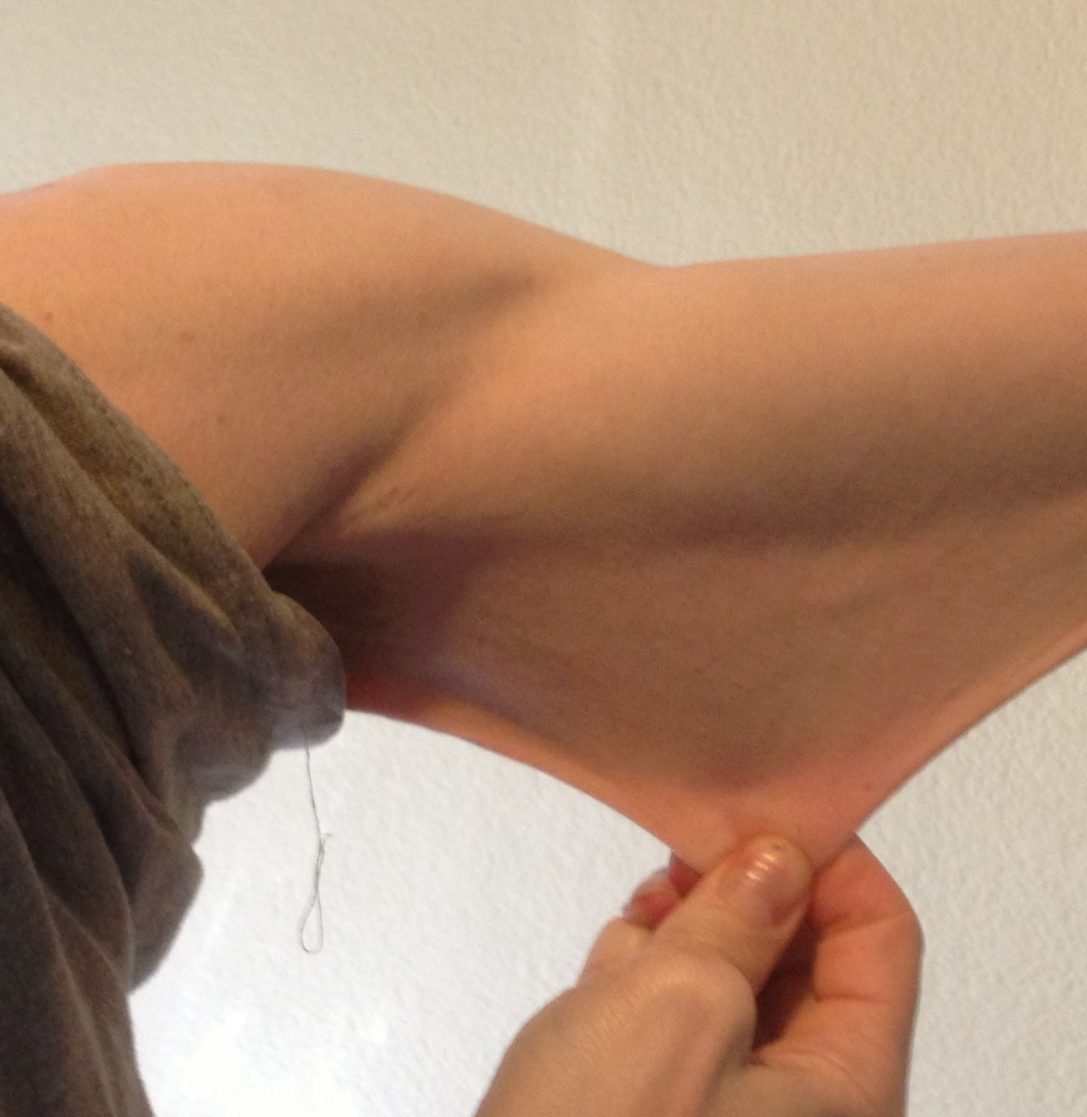
Stretchy skin

I was born in 1971. My childhood was very active. I played soccer and backpacked every summer with my father. There was never any hint of a problem. I have always had a certain degree of hyperflexibility, being able to bend my fingers far backwards (Figures 1-2). My skin, which did not look different from anyone else's, was very stretchy (Figure 3). 

Until 2008, I was very active with a body weight appropriate to my height. I mountain biked and hiked through steep terrain in the back-country. I easily covered great distances carrying a heavy backpack. I had a healthy appetite with no problems. Life was normal and active.

In 2008, I took a two-week road trip across the United States. I was seated for several hours each day. I became constipated. I did not think much of it because I thought it was due to lack of exercise. However, after the trip, the constipation continued and became more and more severe. I was able to have a bowel movement only once every 10 days. A pain associated with eating began and progressively intensified. I began to avoid eating for days at a time just to keep the pain at bay. However, I would become so hungry that I would overeat, become very nauseated, and vomit. Sometimes, I would vomit food eaten the day before.

**Figure 4:**
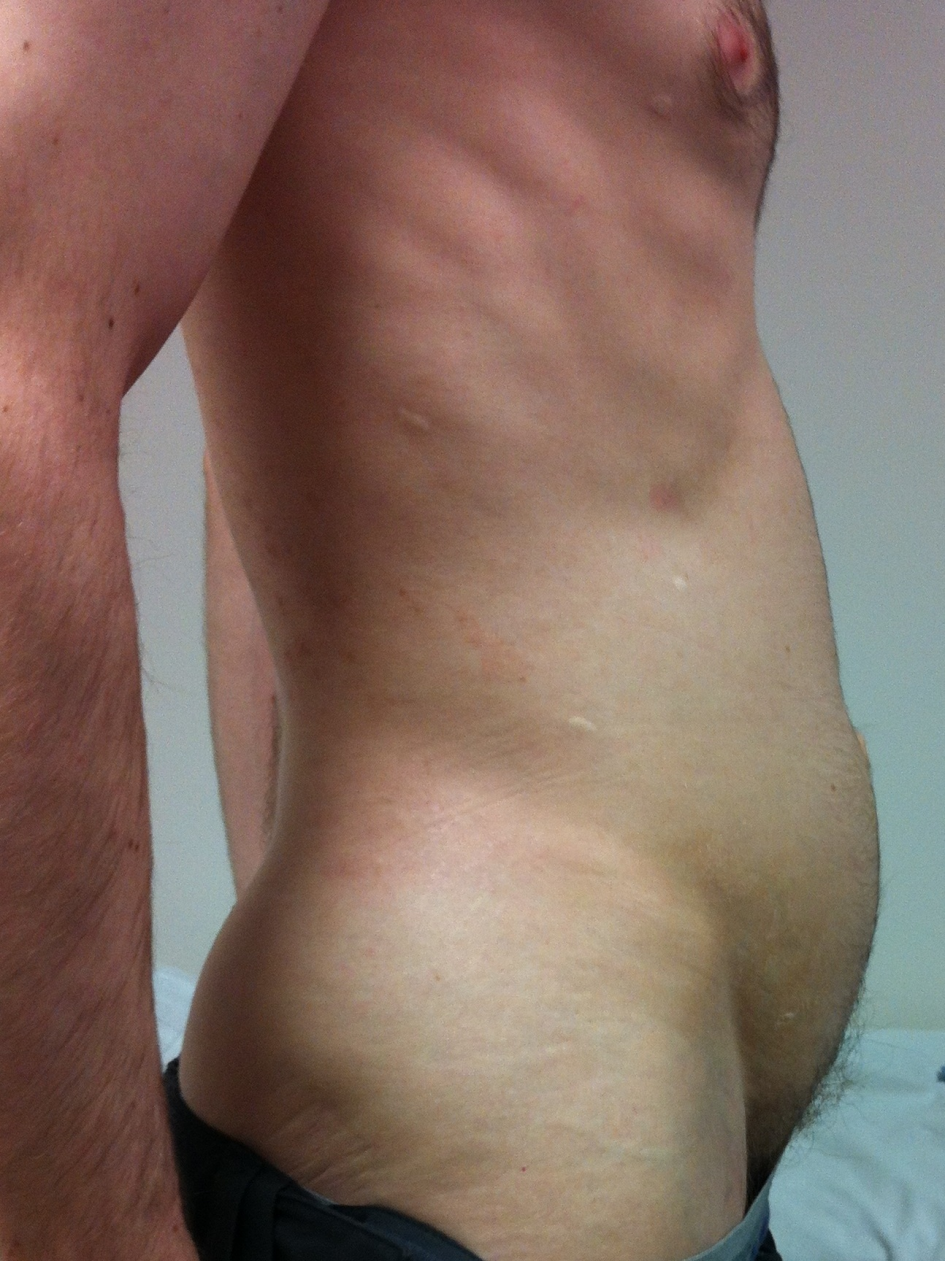
Bloating

My abdomen was very bloated when standing (Figure 4) and I experienced pain in my lower abdomen and also up behind my liver that I can only describe as a tearing, throbbing, pinching sensation. My stool, when I did have a bowel movement, was a weird narrow shape and it was very painful right after the bowel movement.

It is hard to pinpoint when the really severe nausea started. It could have been 2009 or 2010, but the pain, coupled with the nausea, continued to increase. I used to weigh 180 pounds. During this illness, I dropped to 117 pounds.

After my first surgery to look after my slow stomach emptying (details later), the pain and nausea were amplified. When I tried to eat, sometimes the nausea and abdominal pain became very intense. After my second surgery (details later), I was no longer constipated because I now had an ileostomy but...

Today I am in pain all of the time, especially if I try to eat. Even drinking a cup of clear liquid has been painful. I have a hard time sleeping and it feels like a pinching, tearing, throbbing, and intense pressure mostly in the lower abdomen. I would be grateful to have anyone's thoughts on anything that could lead to a better quality of life.

The doctor's story

I first met the patient in 2013 when he was 41 years old. His vomiting had become severe and he had been hospitalized with hypokalemic, hypochloremic, metabolic alkalosis (K^+^ 2.0 mEq/L, Cl^-^ 75 mEq/L, ^-^HCO_3_ 43 mEq/L, pH 7.50). History revealed vomiting of day old food and pills. On examination, his pupils reacted normally to light and there was no postural hypotension or tachycardia. His abdomen was flat when lying and protuberant when standing (Figure 4). There was a loud gastric succussion splash. I did not recognize that he had EDS-HT. Some months later, after much literature searching and puzzling on my part, I asked him to show me what he could do with his fingers (Figures 1-2). As they say, the penny dropped. Two years later, I met his sister who had the same degree of joint hypermobility but fortunately no gastrointestinal symptoms. No other members of the family are known to have EDS.

**Figure 5:**
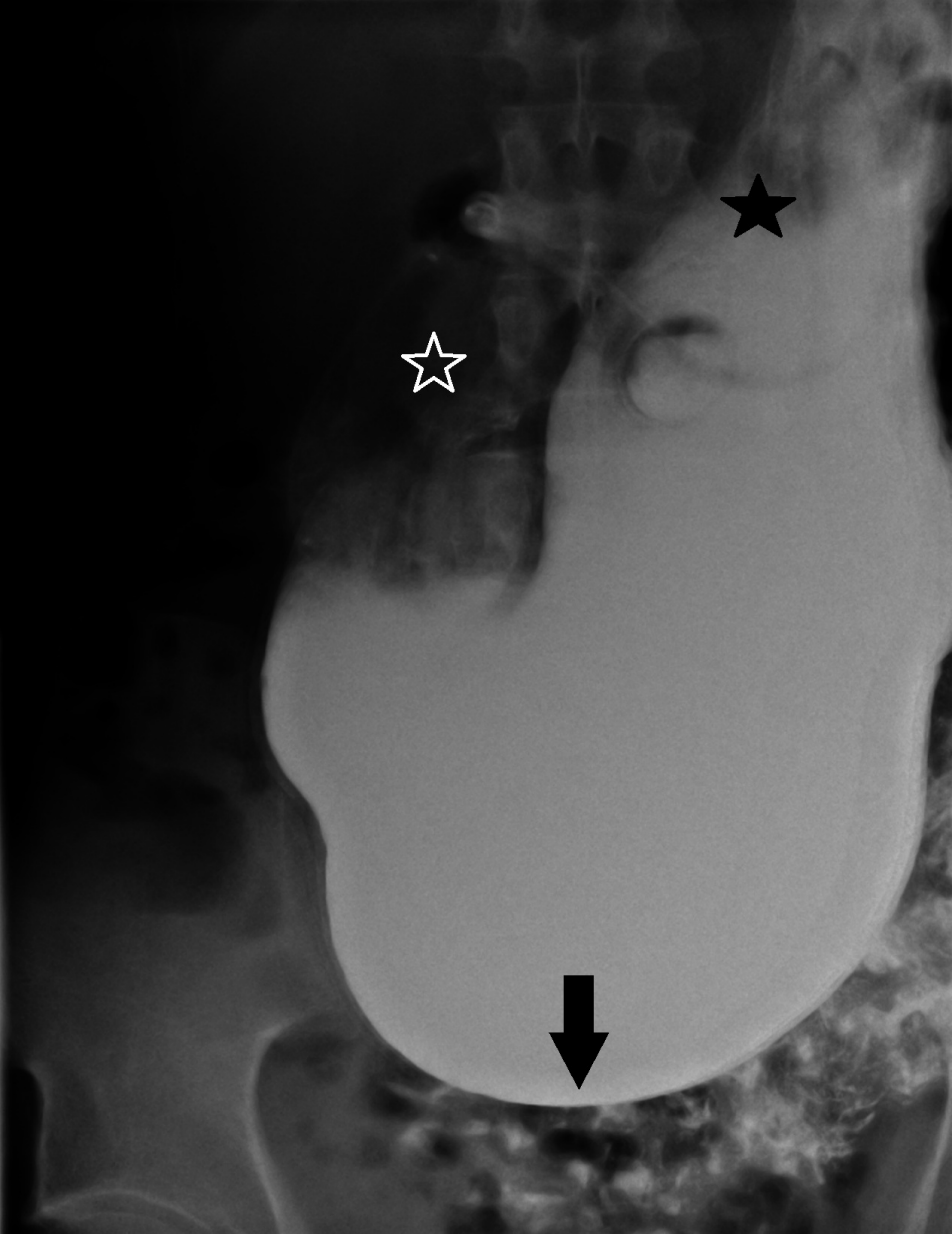
Gastroptosis Arrow = greater curvature of stomach in pelvis; white star = gastric antrum; black star = gastric fundus

**Figure 6:**
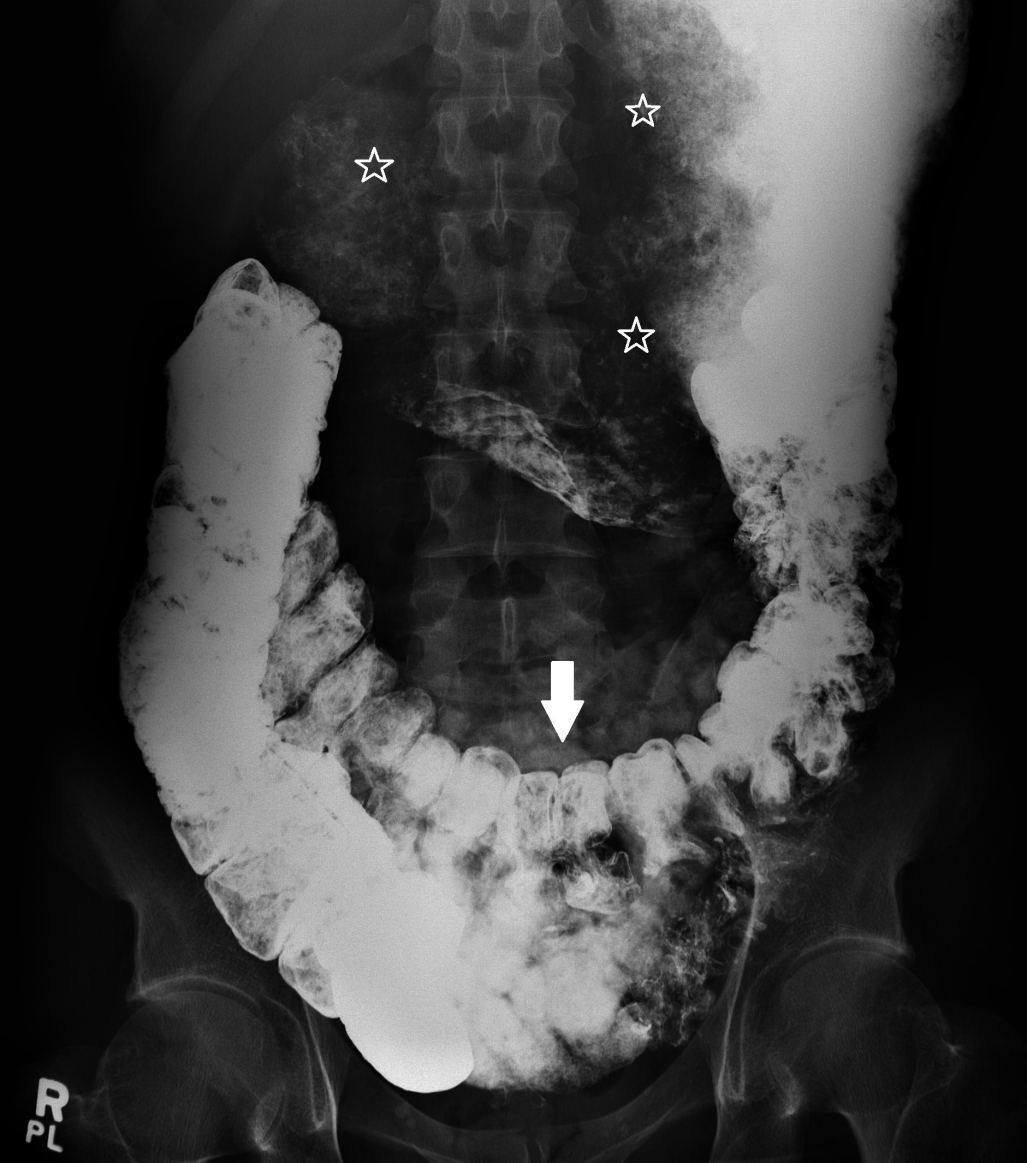
Colonic ptosis Arrow = transverse colon in pelvis; stars = food residue and barium in stomach

**Figure 7:**
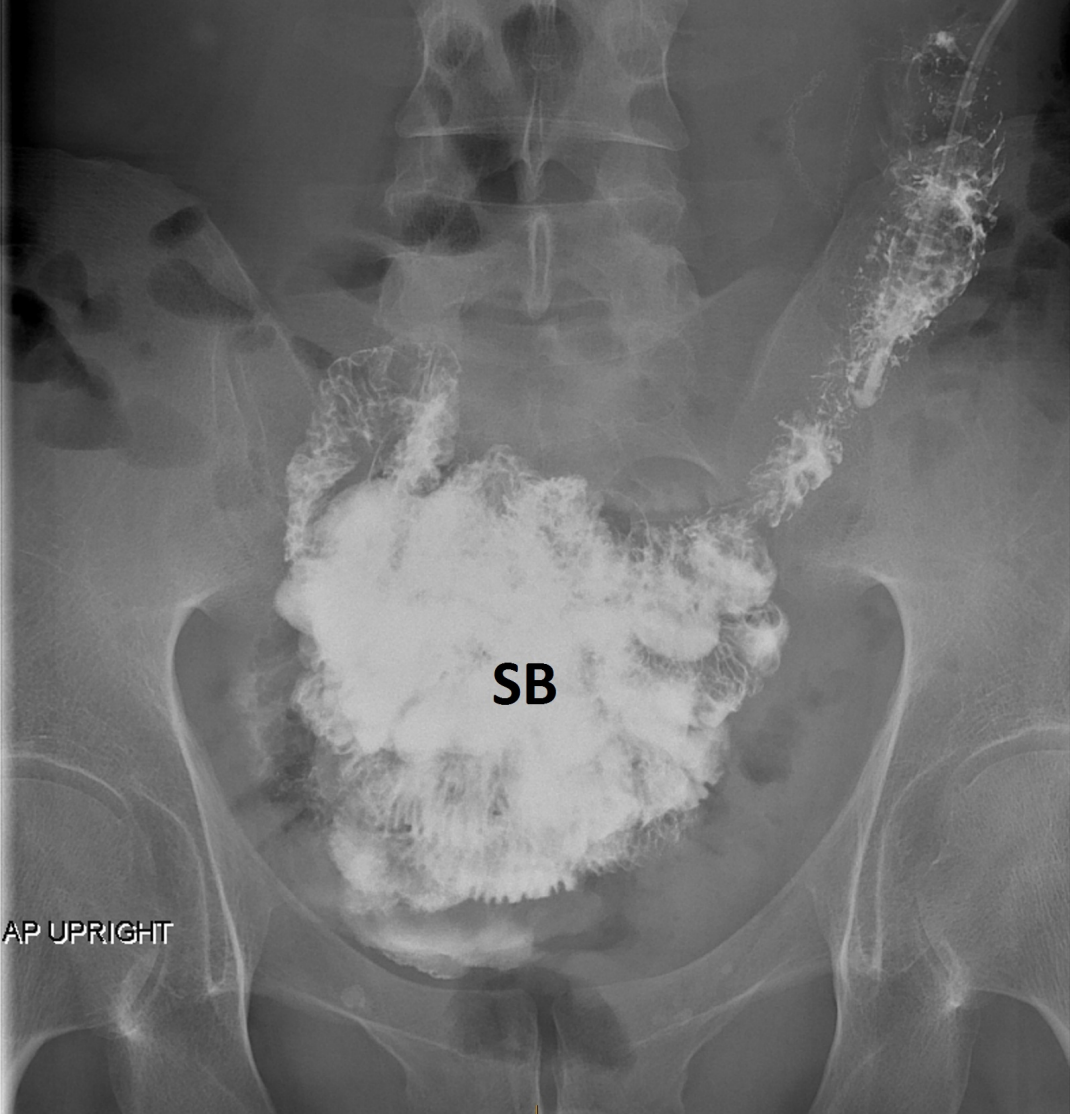
Small bowel in pelvis

**Figure 8:**
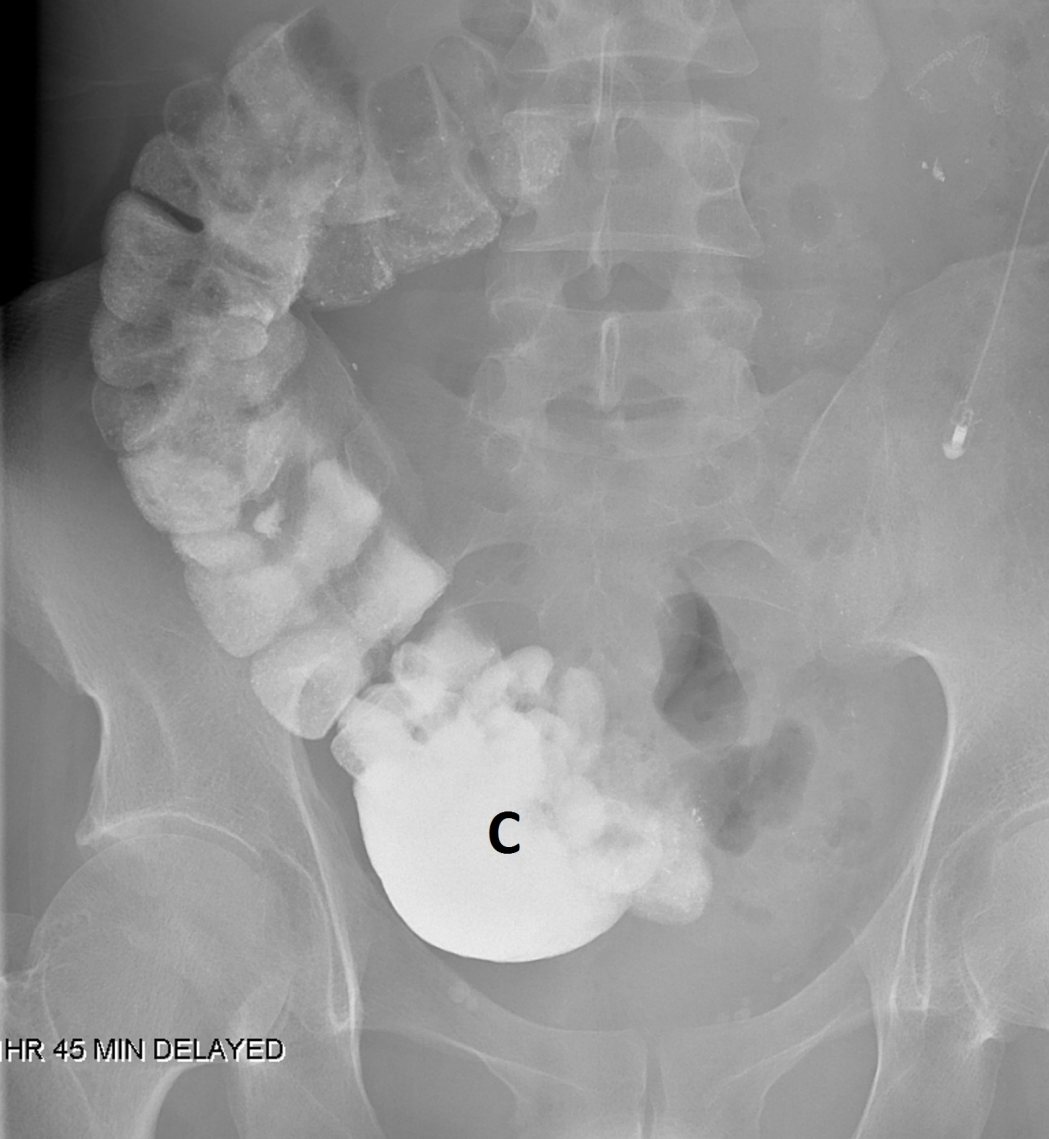
Cecum in pelvis

A barium study of his upper gastrointestinal (GI) tract showed marked gastroptosis (Figure 5). The radioisotope gastric emptying half-time was 190 minutes (normal = 90 - 120 minutes). During a whole gut radio-opaque marker study, 13 of the 19 identified markers were still in his stomach at 23 hours and all 19 markers were still in his rectum and descending colon at seven days. Computerized tomographic (CT) angiography showed a very tight aortic/superior mesenteric artery angle of 9 - 10 degrees (normal 25 - 60 degrees). The superior mesenteric artery (SMA) syndrome was considered, but a dedicated barium study of his upper GI tract showed only the impression of the SMA across the third part of the duodenum without proximal dilatation of the duodenum. Later, the study of an upright film, after he had eaten a sandwich with some barium, showed a lot of material in his stomach as well as barium in his colon and a very "ptotic" transverse colon (Figure 6). A defecating proctogram showed normal emptying but with prominent rectal folds and some small bowel loops posterior to the rectum. CT, barium, and transit studies of the small bowel were normal except that the majority of the loops were deep within the pelvis along with the cecum (Figures 7-8).

A colonoscopy and upper endoscopy were normal, but duodenal biopsies showed intraepithelial lymphocytes compatible with celiac disease. However, tissue transglutaminase antibodies and human leucocyte antigens (HLA) - DQ2 and DQ8 were negative, and a gluten-free diet did not help his symptoms.

Although there was no apparent relationship between his urinary function and his abdominal symptoms, he did have urinary frequency and nocturia. While hospitalized, he once had an episode of gross hematuria. Cystoscopy showed only mild prostatic hypertrophy and mild bladder trabeculation. Needless to say, there were numerous other negative investigations searching for an explanation for his symptoms.

In addition to constipation, weight loss, and vomiting, he had three abdominal pains: right lateral lower abdomen, right lower lateral chest and upper abdomen, and a deep pelvic pain as well as a pain deep to his sacrum. All were described with words such as "jamming, sticking, sharp, tearing, needling, hooking, poking, sharp, and deep". Eating tended to make them all worse. Vomiting and having a bowel movement tended to help as did lying in a Trendelenburg position or using an inversion table. An abdominal support garment did not help. Celiac, splanchnic, and inferior hypogastric plexus blocks did not help nor did infiltration of trigger points in the abdominal and chest walls. A caudal epidural injection of triamcinolone, saline, and lidocaine helped some of the sacral pain. Later local injections of 2% lidocaine diluted 1:10 with saline helped several very painful abdominal wall pains, which developed after his second surgery.

We tried all standard laxatives, enemas, suppositories, metoclopramide, domperidone, erythromycin, proton pump inhibitors, prucalopride, linaclotide, a range of antidepressants and atypical antipsychotics, and even cannabis oil, in an attempt to control his symptoms. They all failed. Feeding by nasojejunal and gastrojejunal tube was tried but made his nausea and lower abdominal pain worse. He required parenteral feeding to maintain nutrition. After 11 months of unsuccessful medical therapy, a 6 x 6 cm proximal gastric pouch and gastrojejunostomy were created laparoscopically. To prevent bile reflux, a side-to-side enteroenterostomy was created between the afferent and efferent limbs of the jejunal loop 15 and 30 cm remote from the gastrojejunal anastomosis. A feeding jejunostomy tube was placed. The vomiting stopped, but abdominal pain and nausea persisted, occurring even with jejunal feeding. Parenteral feeding was restarted. After a further 10 months of attempts to control his symptoms, a partial right colectomy with end ileostomy was performed laparoscopically. There was no histologic evidence of enteric myopathy or neuropathy in the surgical specimen. The constipation "resolved", but the nausea and abdominal pain continued, being worse any time he tried to eat. He is maintained on total parenteral nutrition with occasional small sips by mouth.

## Discussion

The structural gastrointestinal complications associated with the "classic" form of EDS include hernias, intestinal diverticulosis, and rectal prolapse. The occasionally catastrophic complications of the "vascular" form of EDS include hemorrhages and intestinal perforations [1]. However, by far, the commonest form of EDS is the hypermobility type (EDS-HT), also called the joint hypermobility syndrome (JHS). It is inherited in an autosomal dominant manner, and diagnosis is based entirely on clinical findings and family history. It has an extremely strong association with the so-called  "functional" symptoms of dyspepsia, reflux, bloating, nausea, vomiting, diarrhea, constipation, and chronic abdominal pain [2]. When investigated further, gastroparesis, esophageal dysmotility, abnormal small bowel motility, slow transit constipation, rectal defecatory dysfunction, small bowel dilatation, and even celiac disease may be found [3-5]. We can find only two full case reports [6-7] and a few brief mentions with illustrative pictures of visceroptosis associated with EDS in the medical literature [5, 8-9].

In addition to functional and structural gastrointestinal abnormalities, patients with EDS-HT/JHS may be plagued by chronic unexplained pains, poor sleep, profound fatigue, and symptoms of dysautonomia. A recent study found that many have a small fiber neuropathy suggesting that their symptoms may have a neuropathic rather than a "structural" origin [10]. Perhaps the pain is unrelated to the visceroptosis. 

## Conclusions

Although considered "benign", EDS-HT/JHS can be a devastating disorder that causes severe physical and psychological distress and marked functional disability.

None of the antinauseants and prokinetics we tried helped. Of all the laxatives, only polyethylene glycol (PEG) worked but at the cost of worsening abdominal pain and nausea. Narcotics helped keep the pain tolerable but made constipation and fatigue worse.

Does surgery have a role? The scant literature provides no answer and suggests that any benefit may be only temporary. Dr Marco Castori wrote us that in his large EDS clinic, of the few patients who had GI surgery, "none experienced long-lasting beneficial effects and many developed worsening of their general health status".  In the case we are reporting, the gastric pouch and gastrojejunostomy did stop the vomiting and the ileostomy did remove constipation from the equation, but the patient is still in constant pain which worsens whenever he tries to eat.

What to do now? Small bowel transplantation has been suggested.
